# Prevention and management of dental erosion and decay

**DOI:** 10.1186/s12903-024-04257-y

**Published:** 2024-04-17

**Authors:** Guglielmo Campus, John Yun Niu, Berkant Sezer, Ollie Yiru Yu

**Affiliations:** 1 Klinik für Zahnerhaltung, Präventiv- und Kinderzahnmedizin Zahnmedizinische Kliniken, University of Bern, Bern, Switzerland; 2 Faculty of Dentistry, The University of Hong Kong, Hong Kong, China; 3 Department of Pediatric Dentistry, School of Dentistry, Çanakkale Onsekiz Mart University, Çanakkale, Türkiye

**Keywords:** Dental caries, Dental erosion, Management, Prevention, Tooth decay

## Abstract

The 2017 Global Disease Study revealed 2.3 billion untreated cavities and 139 million other oral conditions like dental erosion. Modern treatments prioritise controlling etiological factors and preventing related diseases. This Editorial invites researchers to contribute to the collection, *‘Prevention and management of dental erosion and decay’*.

Dental caries is considered a lifelong non-communicable disease, affecting people from early childhood to late adulthood. Dental caries results from cariogenic microorganisms converting free sugars in food and drinks into acids, damaging dental tissues over time, and significantly affects the oral and general health and quality of life of individuals, families, and societies. Although it is a largely preventable disease, its prevalence has not changed significantly in the last 30 years and continues to be a threat to public health [1]. In light of scientific developments, new paradigms in the conceptualization of dental caries have emerged in recent years, causing changes in both preventive and therapeutic perspectives. Dental caries, which has long been described as transmissible and infectious, is now described as ecological, non-infectious, and preventable [2]. It is known to be associated with dysbiosis in the dental biofilm and occurs due to exposure to free sugars. However, it is strongly linked to harmful lifestyles and behaviors, mainly associated with an improper diet [1–3]. Accurate knowledge of the characteristics of dental caries is important for developing new approaches to managing dental caries in order to raise caries-free generations and prevent tooth decay.

Dental erosion, or erosive tooth wear, is another dental hard tissue disease that negatively impacts individuals’ well-being. It involves the progressive loss of dental tissue due to physical or chemo-physical processes, emphasizing the role of chemical acids [4]. Patients with erosive tooth wear may experience dentin hypersensitivity, functional tooth damage, and worsening aesthetic appearance marked by discoloration or shortening of teeth. A significant connection was found between tooth wear and declining oral health-related quality of life among participants [5]. Erosive tooth wear prevalence varies across regions. Selective studies have reported the prevalence of erosive tooth wear in adults, ranging from 2 to 100% across different areas [5–7]. However, data on the prevalence of erosive tooth wear remains limited or unavailable in many countries in Asia, North America, South America, Southeastern Europe and Africa [5]. The severity of erosive tooth wear increases with age [8]. The seriousness of erosive tooth wear has been growing among adults, particularly older adults, with a considerable percentage of patients experiencing moderate or severe stages. Dentin involvement was observed in 2–45% of erosive tooth wear cases [5]. Erosive tooth wear is a cumulative, irreversible process. Without proper management strategies, tooth wear may continue to progress. Moderate or advanced stages of erosive tooth wear necessitate restorative treatments, which typically involve multiple teeth or the entire arch and can be complicated and expensive. Economically disadvantaged and socially deprived older adults may not be able to afford such treatments. Therefore, practical and innovative management and prevention approaches are crucial to slowing dental erosion progression and minimizing the need for complex and costly restorative treatments [9].

Managing common diseases involving dental hard tissues, such as dental caries and dental erosion, is quite complex. Modern approaches to these diseases should primarily include the prevention of diseases by prioritizing the principle of preventability. In addition, it is important to include the management of these diseases, which are described as non-communicable and preventable, in public health policies in the context of public oral and dental health. Besides established evidence-based oral care routines such as regular tooth brushing with fluoridated products, additional measures based on ecological principles may address biofilm dysbiosis [3]. Methods to reduce sugar intake, slow down plaque metabolism, and improve salivary characteristics should be considered in terms of effectiveness, compliance, and cost-effectiveness [1–3]. Furthermore, biofilm engineering early in life through prebiotics and probiotics to support the acquisition of a balanced microbiome appears to be a promising approach to achieving a long-term anti-decay effect [3].

Due to the current understanding of dental caries, the contemporary view of caries treatment options has shifted from the traditional approach to a novel concept more oriented toward controlling etiological factors [10, 11]. These approaches are also compatible with minimally invasive dentistry, which aims to preserve tooth structure and pulp vitality and thus prolong the life of teeth. The main aim of this approach is to cure the disease by improving oral health. Although traditional caries removal methods and treatment options are still preferred in many populations today, approaches are changing towards non-invasive methods with developments in adhesive dentistry and a better understanding of the behavior of dental caries. Similarly, managing dental erosion can be challenging, invasive, and extensive. Non-restorative approaches to dental erosion often include dietary analysis and counselling, oral health education, and topical use of anti-erosive agents. Additionally, among various non-restorative approaches, topical anti-erosive agents are widely used to treat dental erosion [9]. Restorative management is indicated in cases where tooth integrity is threatened, aesthetics is impaired, dentin hypersensitivity exists, and pulp exposure is likely. Currently, there are various options for treating tooth wear, ranging from conservative (adhesive and composite resin restorations) to more invasive procedures (crowns, bridges, and even full-mouth reconstruction).

Because dental erosion and dental caries are among the oral and dental diseases that affect individuals most frequently, and due to their importance in the oral and dental health of society, as the editors of this collection, we invite researchers to enrich this collection with their articles.

## Data Availability

No datasets were generated or analysed during the current study.
